# Generating tumor-specific T cells based on a head and neck cancer organoid for adoptive cell therapy

**DOI:** 10.3389/fimmu.2025.1573965

**Published:** 2025-06-12

**Authors:** Yinyu Chen, Shoupeng Wang, Luxi Zheng, Lin Chen, Feng Xu, Shuqi Guo, Jian Meng

**Affiliations:** ^1^ Department of Stomatology, Xuzhou Central Hospital, Xuzhou, Jiangsu, China; ^2^ School of Stomatology, Xuzhou Medical University, Xuzhou, China; ^3^ The Xuzhou Clinical College of Xuzhou Medical University, Xuzhou Medical University, Xuzhou, China

**Keywords:** adoptive cell therapy, peripheral blood T cells, head and neck cancer, organoid, co-culture

## Abstract

**Background:**

A head and neck cancer organoid (HNCO) and peripheral blood T cell co-culture model was established to investigate whether HNCOs can induce the differentiation of peripheral blood T cells into tumor-reactive T cells. Additionally, this study seeks to explore the cytotoxicity of these T cells against autologous tumor organoids, providing theoretical and experimental evidence for the feasibility of this model as a platform for adoptive cell immunotherapy in head and neck cancer (HNC).

**Methods:**

HNCO single cells were co-cultured with peripheral blood lymphocytes (PBLs) collected and isolated from patients with HNC. The culture supernatant was collected and assayed for interferon-gamma (IFN-γ) and tumor necrosis factor-α (TNF- α). The expression of T cell activation markers cluster of differentiation (CD)137 and CD107a was measured by flow cytometry to confirm tumor specificity and cytotoxicity. Additionally, the optimal effector-to-target (E/T) ratio was determined using the Cell Counting Kit-8 assay, and HNCO killing was quantified by fluorescent labeling.

**Results:**

Of the 27 successfully established HNCO-T cell co-culture systems, 81.48% induced the *in vitro* differentiation and tumor-reactive CD8^+^ T cell expansion capable of mediating the killing of mature HNCOs.

**Conclusion:**

The patient-derived HNCO-T cell co-culture model effectively induced PBL differentiation into tumor-reactive CD8^+^ T cells with enhanced tumor-killing activities. This model serves as a novel *in vitro* preclinical tool for advancing personalized adoptive immunotherapy in HNC.

## 1 Introduction

According to the Global Cancer Database, HNC is highly prevalent among men in developing countries, particularly in Central and South Asia, with its mortality ranking third among all cancers (1, 2). HNC is a heterogeneous disease affecting multiple sites in the head and neck region. Surgery, chemoradiotherapy, and other combination treatments have substantially halted disease progression and prolonged patient survival; nonetheless, they are often associated with adverse effects that worsen the patient’s quality of life (3). This necessitates safe and effective novel treatments for the clinical diagnosis and treatment of HNC.

Immunotherapy has emerged as a promising treatment modality for cancer. Adoptive T-cell therapy (ACT) is a highly personalized form of tumor immunotherapy that involves culturing the patient’s autologous T cells in the laboratory and reinfusing them into the patient to mediate immune responses (4–6). T-cell cytotoxicity is critical for effective immunotherapy. During *in vitro* culture, T cells can be specifically stimulated to differentiate into cytotoxic cluster of differentiation (CD)8^+^ T lymphocytes (CTLs), which mediate the recognition and killing of tumor cells. A higher proportion of CD8^+^ T cells is associated with a more robust response to tumor cells.

Three major modalities of ACT exist: tumor-infiltrating lymphocytes (TILs), chimeric antigen receptor T cells (CAR-T), and T cell receptor-engineered T cell (TCR-T) therapies. TIL therapy involves expanding heterogeneous populations of endogenous T cells from tumor tissues (7), whereas CAR-T and TCR-T therapies focus on expanding genetically engineered T cells that target specific antigens (8). ACT has demonstrated impressive clinical responses in hematologic malignancies; nonetheless, its application in solid tumors is under investigation (9, 10). However, HNCs are highly immunosuppressive because of the low number of T cells in the tumor microenvironment and inflammatory infiltrates that render existing TILs ineffective. This makes it difficult to obtain adequate tumor-reactive TILs from patients with HNC for ACT (11–13). Therefore, generating immune cells that can specifically recognize and kill tumor cells *in vitro*, as well as enhancing their anti-tumor responses, are key areas of intensive research. Upon reaching a sufficient quantity, these *in vitro*-cultured immune cells can be reinfused into the patient, where they mediate therapeutic effects as a cellular drug. Immune cell reinfusion exerts less impact on healthy tissues, compared with chemotherapy and other agents that directly kill cancer cells.

To obtain specific tumor-reactive immune cells *in vitro*, autologous peripheral blood lymphocytes (PBLs) were isolated from patients and used as the source of T cells. These cells were co-cultured with primary HNCOs in a three-dimensional culture. HNCOs effectively simulate the tumor microenvironment and accurately recapitulate tumor characteristics observed in patients, thereby supporting in-depth research on HNC and providing data for the development of personalized treatment regimens (14–16). The use of HNCOs helps to advance the understanding of tumor-immune interactions, which is crucial for tumor immunotherapy research and the clinical translation of personalized immunotherapy.

This study seeks to investigate the antitumor response of T cells derived from the organoid co-culture system by measuring cytokine secretion and the expression of cytotoxic markers.

## 2 Materials and methods

### 2.1 Sample source

Patients with HNC were recruited from the Xuzhou Central Hospital. The study protocol was reviewed and approved by the Biomedical Research Ethics Committee of Xuzhou Central Hospital. Informed consent was obtained from all patients and their families before specimen collection. The pathological type of the HNC samples was confirmed by the Pathology Department of Xuzhou Central Hospital before further processing.

### 2.2 Materials

Human fibroblast growth factor 10, recombinant human R-spodin-1 protein, recombinant human noggin protein, recombinant human Wnt-3A protein, N-acetylcysteine, A83-01, gastrin I, and nivolumab were purchased from MedChemExpress (Monmouth Junction, USA). Advance Dulbecco’s Modified Eagle’s Medium/F12 basal medium, Roswell Park Memorial Institute-1640 medium, N-2-hydroxyethylpiperazine-N’-2-ethanesulfonic acid buffer, GlutaMAX™, B27 supplement, and N2 supplement were purchased from ThermoFisher (Waltham, MA USA). Nicotinamide, protablin E 2, and butylhydroxyanisole were purchased from Sigma. Rock Inhibitor (Y-27632) was purchased from Selleck Chemicals (Houston, USA). Human epidermal growth factor and recombinant human interleukin-2 were purchased from Peprotech (New Jersey, USA). Penicillin/streptomycin/amphotericin solution was purchased from Beyotime Biotech Inc. (Shanghai, China).

### 2.3 Establishing the HNCO model

Fresh HNC tissues—obtained by biopsy or surgical resection—were transported to the laboratory for organoid culture. Tumor tissue was minced and digested into a single-cell suspension. Specifically, epithelial-origin tumors were digested with collagenase I, whereas salivary gland tumors were digested with collagenase II. Single-cell suspensions were cultured in Matrigel to form organoids resembling the original tumors. The culture medium was changed twice weekly, and the organoids were passaged approximately every 2 weeks (17, 18).

### 2.4 Histology

Mature HNCOs were collected, centrifuged, and resuspended in liquid agarose. Once solidified, the organoids were dehydrated, cleared, embedded in paraffin, sectioned, and stained with hematoxylin and eosin (H&E). They were processed for immunohistochemistry (IHC) and mounted. The organoids were compared with the original tumor sections.

### 2.5 PBL isolation and T cell expansion

Fresh whole blood samples were collected and transported to the laboratory. The peripheral blood samples and tumor specimens used for co-culture model establishment were obtained from the same patients. PBLs were isolated using density gradient centrifugation with a lymphocyte separation solution under sterile conditions. After cell counting and viability assessment, PBLs were cultured in a T cell expansion medium at 37°C with 5% CO_2_ overnight.

### 2.6 Co-culture of T cells with HNCO single cells

HNCO single cells were collected, counted, and stimulated with interferon-gamma (IFN-γ) for 24 h before co-culture. Coated U-bottom 96-well plates with 5 μg/mL CD28 antibody.Isolated T cells were counted, mixed with HNCO single cells at a predefined effector-to-target (E/T) ratio, and seeded into 96-well U-shaped culture plates for co-culture, cultured in presence of 150 U/mL IL-2 and 20 μg/mL Nivolumab. T cells co-cultured with HNCO for 14 days were labeled as “CT,” whereas those at day 0 of co-culture were labeled as “UT.”

### 2.7 Enzyme-linked immunosorbent assay

Cytokine release from *in vitro*-activated T cells was quantified using the Human TNF-α and Human IFN-γ enzyme-linked immunosorbent assay (ELISA) kit (19, 20). Co-culture supernatants were collected, and mean IFN-γ and TNF-α concentrations were calculated from OD450 values measured by a microplate reader using a standard curve.

### 2.8 Flow cytometry

T-cell markers CD137 and CD107a were quantified using flow cytometry (21–24). Briefly, T cells were re-stimulated with single cells for 5 h, stained with anti-CD3-PerCP-Cy5.5, anti-CD4-FITC, anti-CD8-BV421, anti-CD137-APC, CD107a-PE, and near-infrared viability dye, and analyzed using a flow cytometer. Data were processed using FlowJo software (25).

### 2.9 Cell counting kit-8 assay

To determine the optimal E/T ratio, T cells were collected, counted, resuspended in a culture medium, and adjusted to a predefined density relative to HNCO (per single cell count). They were seeded into 96-well plates. After 24 h, diluted Cell Counting Kit-8 (CCK8) reagent was added, and the cells were incubated at 37°C for 2 h. OD450 was measured using a microplate reader to quantify viable tumor cells in each well.

### 2.10 Fluorescent labeling

Cells were stained using the Calcein-acetoxymethyl ester (AM)/propidium iodide (PI) kit, labeling live cells with Calcein-AM and dead cells with PI. Fluorescence was observed under an inverted fluorescence microscope, and fluorescence intensity was quantified using ImageJ.

### 2.11 Data analysis

Statistical analysis was conducted using GraphPad Prism. Independent t-tests were used to compare between two groups, whereas one-way analysis of variance was used to compare among multiple groups. A P-value <0.05 indicated statistical significance.

## 3 Results

### 3.1 Establishment and morphological validation of the HNCO model

**Figure 1:**
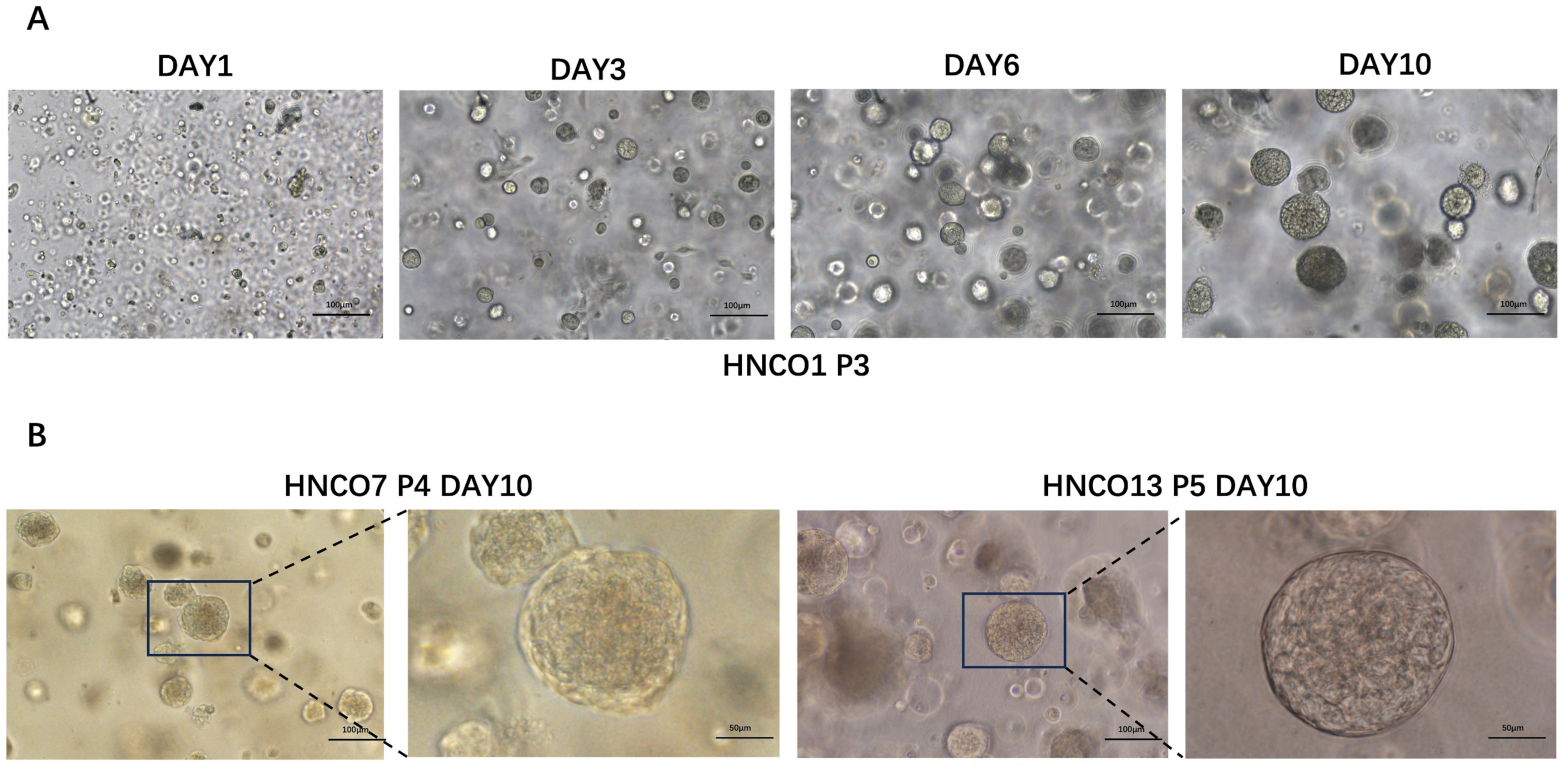
**(A)** Course of HNCO1 growth (100 um). **(B)** Microscopic images of HNCO7 and HNCO13 (100 um, 50 um) HNCO: head and neck cancer organoid.

A total of 27 patient-derived HNCO models were developed. **Table 1** summarizes the clinical pathology data and passage of HNCOs, all enrolled patients were diagnosed with primary head and neck carcinoma and had not received any prior anti-tumor therapies (including radiotherapy, chemotherapy, immunotherapy or biological therapy) for their current tumors. Additionally, none of the patients presented with hematological disorders. HNCOs formed 100 to 150 µm spherical structures after 3 to 5 days of culture and were stably expanded for over four generations without morphological changes (**Figures 1A, B**).

**Table 1:** Patient information.

ID	Gender	Age	Primary site	Clinical TNM stage			Histopathological subtype	Passage
				T	N	M		
HNCO1	Male	66	Tongue	4	0	0	OSCC	5
HNCO2	Female	81	Tongue	2	0	0	OSCC	6
HNCO3	Female	66	Gingiva	2	0	0	OSCC	7
HNCO4	Female	33	Parotid gland	3	0	0	DC	5
HNCO5	Female	56	Sublingual gland	3	0	0	MEC	5
HNCO6	Female	85	Parotid gland	3	2	0	ACC	6
HNCO7	Female	80	Gingiva	2	0	0	OSCC	8
HNCO8	Female	46	Tongue	2	0	0	OSCC	7
HNCO9	Female	81	Cheek mucosa	3	1	0	OSCC	5
HNCO10	Male	82	Gingiva	2	0	0	OSCC	6
HNCO11	Female	93	Cheek mucosa	3	0	0	OSCC	5
HNCO12	Female	57	Tongue	4	1	0	OSCC	5
HNCO13	Male	76	Tongue	3	0	0	OSCC	5
HNCO14	Male	52	Cheek mucosa	2	2b	0	OSCC	7
HNCO15	Male	65	Mouth floor	2	0	0	OSCC	6
HNCO16	Male	71	Maxilla	3	1	0	OSCC	5
HNCO17	Male	66	Gingiva	4b	0	0	OSCC	5
HNCO18	Male	70	Tongue	3	0	0	OSCC	5
HNCO19	Female	61	Tongue	4a	0	0	OSCC	5
HNCO20	Male	77	Maxilla	2	0	0	OSCC	6
HNCO21	Male	51	Gingiva	3	1	0	OSCC	6
HNCO22	Male	72	Gingiva	4a	1	1	OSCC	7
HNCO23	Female	84	Tongue	4	0	0	OSCC	6
HNCO24	Female	77	Tongue	2	1	0	OSCC	6
HNCO25	Female	68	Mouth floor	3	0	0	OSCC	5
HNCO26	Female	67	Mandible	2	0	0	OSCC	6
HNCO27	Female	67	Tongue	3	1	0	OSCC	8
N1	Male	72	Tongue	2	0	0	OSCC	–
N2	Male	74	Tongue	3	1	0	OSCC	–
N3	Female	87	Tongue	2	0	0	OSCC	–
N4	Male	69	Palate	2	0	0	MEC	–
N5	Male	61	Tongue	3	0	0	OSCC	–
N6	Male	51	Tongue	3	1	0	OSCC	–
N7	Female	74	Mouth floor	4	0	0	OSCC	–
N8	Female	69	Parotid gland	2	1	0	ACC	–

OSCC, Oral Squamous Cell Carcinoma; DC, Ductal Carcinoma; MEC, Muccepidermcid Carcinoma; ACC, Adenoid Cystic Carcinoma.

**Figure 2:**
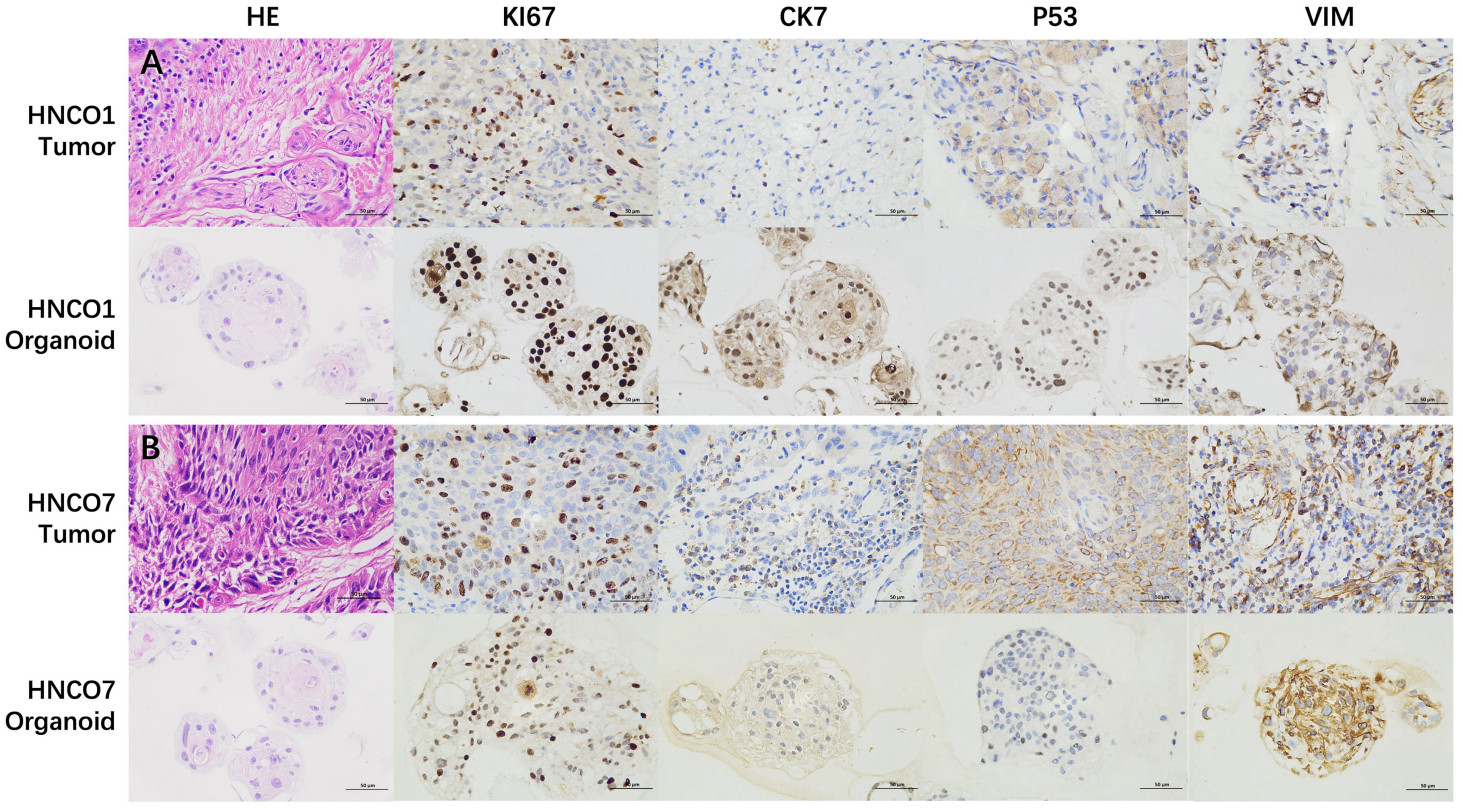
**(A)** H&E and IHC images of HNCO1 vs. parental tumor tissues (50 um). **(B)** H&E and IHC images of HNCO7 vs. parental tumor tissues (50 um). HNCO, head and neck cancer organoid; IHC, immunohistochemistry; and H&E, hematoxylin and eosin.

The morphological and histological characteristics of HNCOs were compared with their parental tumors using H&E staining and IHC analysis. HNCOs closely resembled the parental tumor tissues, particularly in nuclear atypia. Furthermore, IHC analysis indicated canonical tumor markers, including the high expression of pan-cytokeratin, tumor protein p53, and Antigen Kiel 67, confirming the successful establishment of histopathologically consistent HNCOs (**Figures 2A, B**).

### 3.2 Increase in IFN-γ and TNFα after co-culture

**Figure 3:**
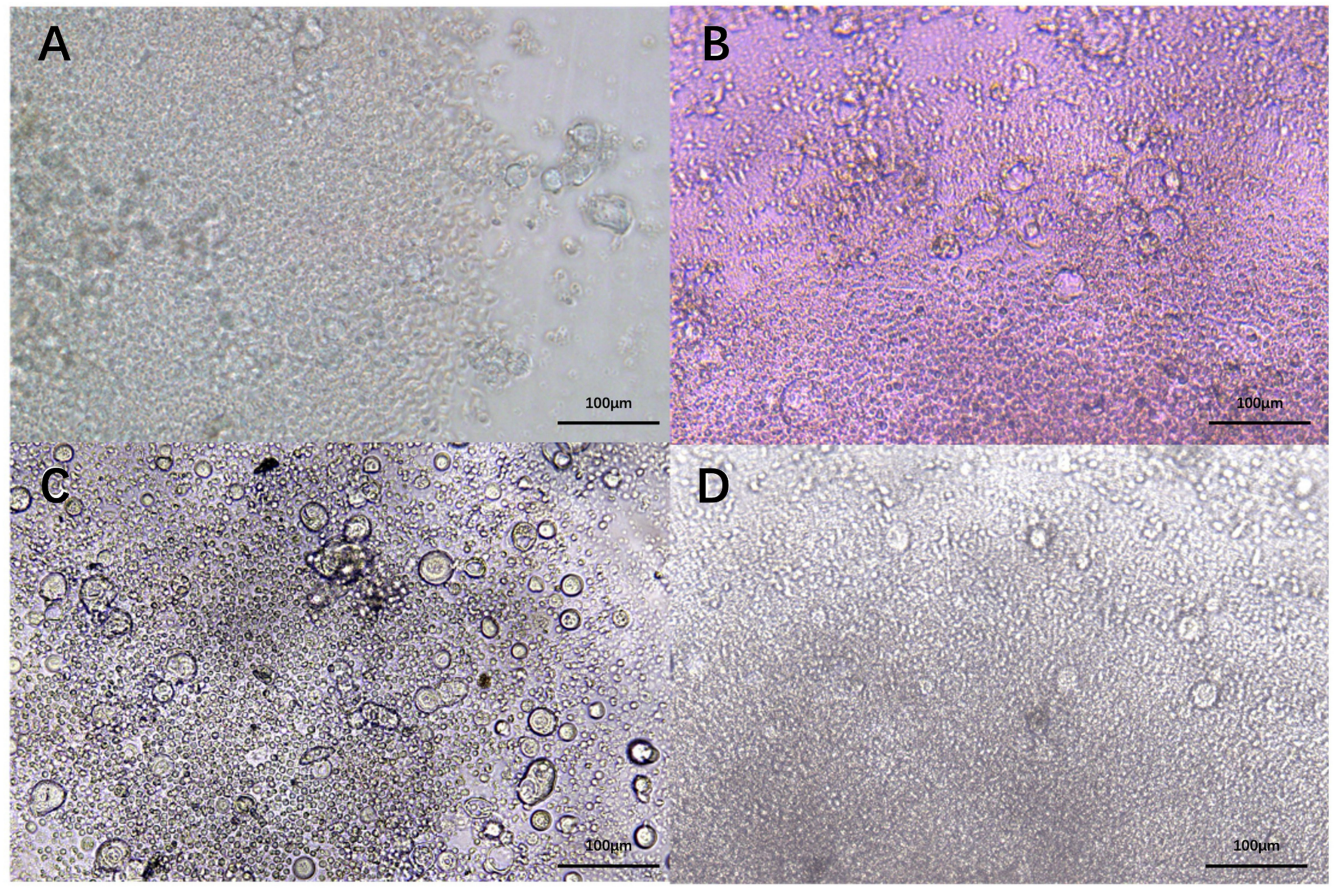
HNCO2 **(A)**. HNCO6 **(B)**, HNCO10 **(C)**, and HNCO12 **(D)** single cells in co-culture with autologous PBLs (100 um). HNCO: head and neck cancer organoid; PBL: peripheral blood lymphocyte.

Effector cytokines IFN-γ and TNFα are central to the activation, differentiation, and tumor-killing activity of CTLs. To assess whether HNCOs could induce T cell-mediated anti-tumor response, single cells from the 27 HNCOs were co-cultured with autologous PBL-derived T cells (**Figure 3**). Before co-culture, HNCO single cells were pre-treated with IFN-γ and nivolumab (anti-PD-1 antibody) to enhance antigen presentation and counteract PD-L1-induced inhibition of T cell activation (26).

**Figure 4:**
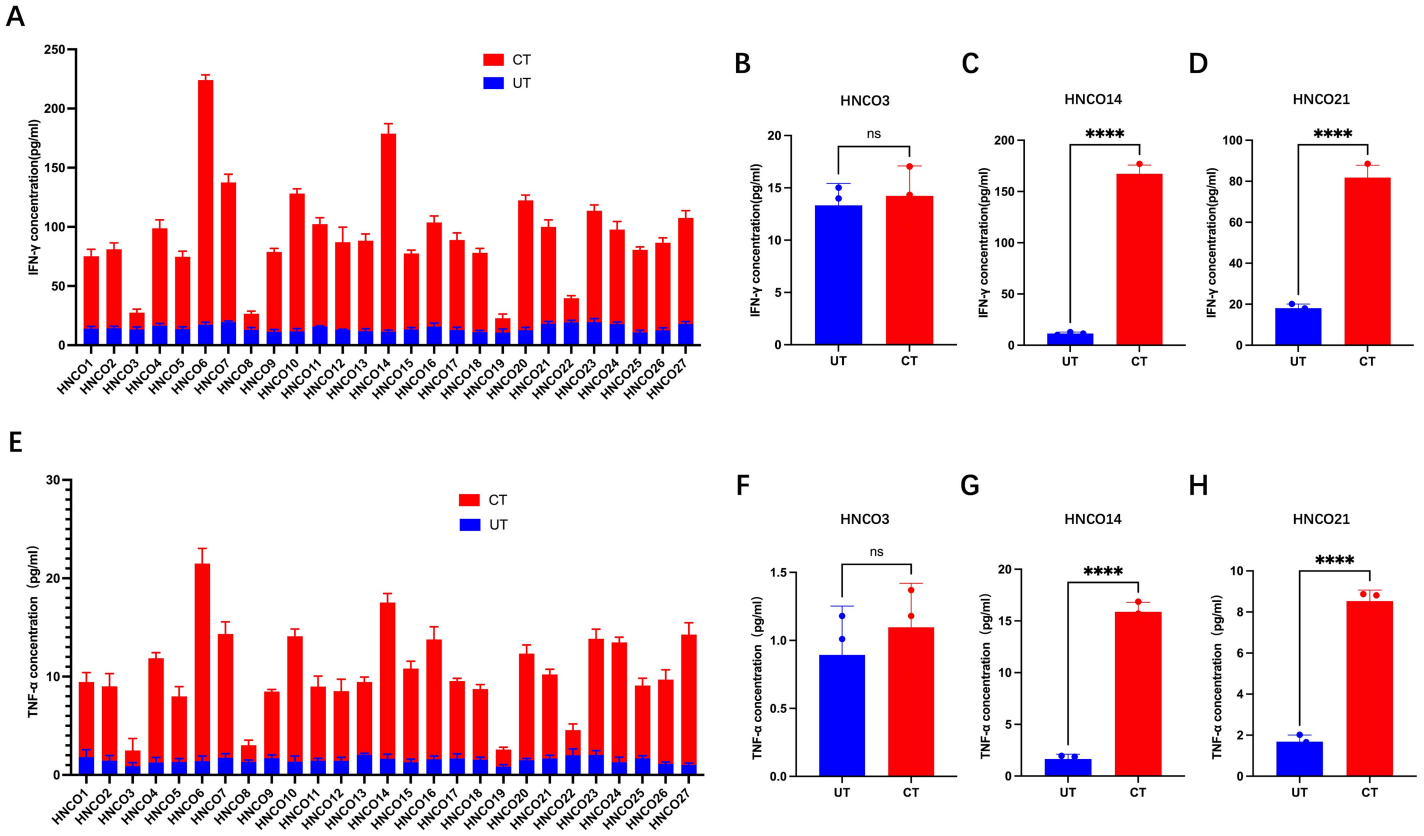
**(A)** IFN-g production in 27 co-culture models. IFN-g levels before and after co-culture with HNCO3 **(B)**, HNCO14 **(C)**, or HNCO21 **(D)**, ns P>0.05, **: P<0.0001. **(E)** TNF-a production in 27 co-culture models. TNF-a before and after co-culture with HNCO3 **(F)**, HNCO14 **(G)**, or HNCO21 **(H)**, ns: P>0.05, **P<0.0001. HNCO, head and neck cancer organoid; IN-g, interferon-gamma; and TNF-a, tumor necrosis factor alpha.

Supernatant samples were collected on day 0 and day 14 to quantify IFN-γ and TNF- α concentrations using ELISA kits. After 14 days of co-culture, IFN-γ and TNF-α levels significantly increased in 23 co-culture systems, whereas remained unchanged in the remaining 4 systems (**Figure 4**).

### 3.3 T cell activation and tumor reactivity

**Figure 5:**
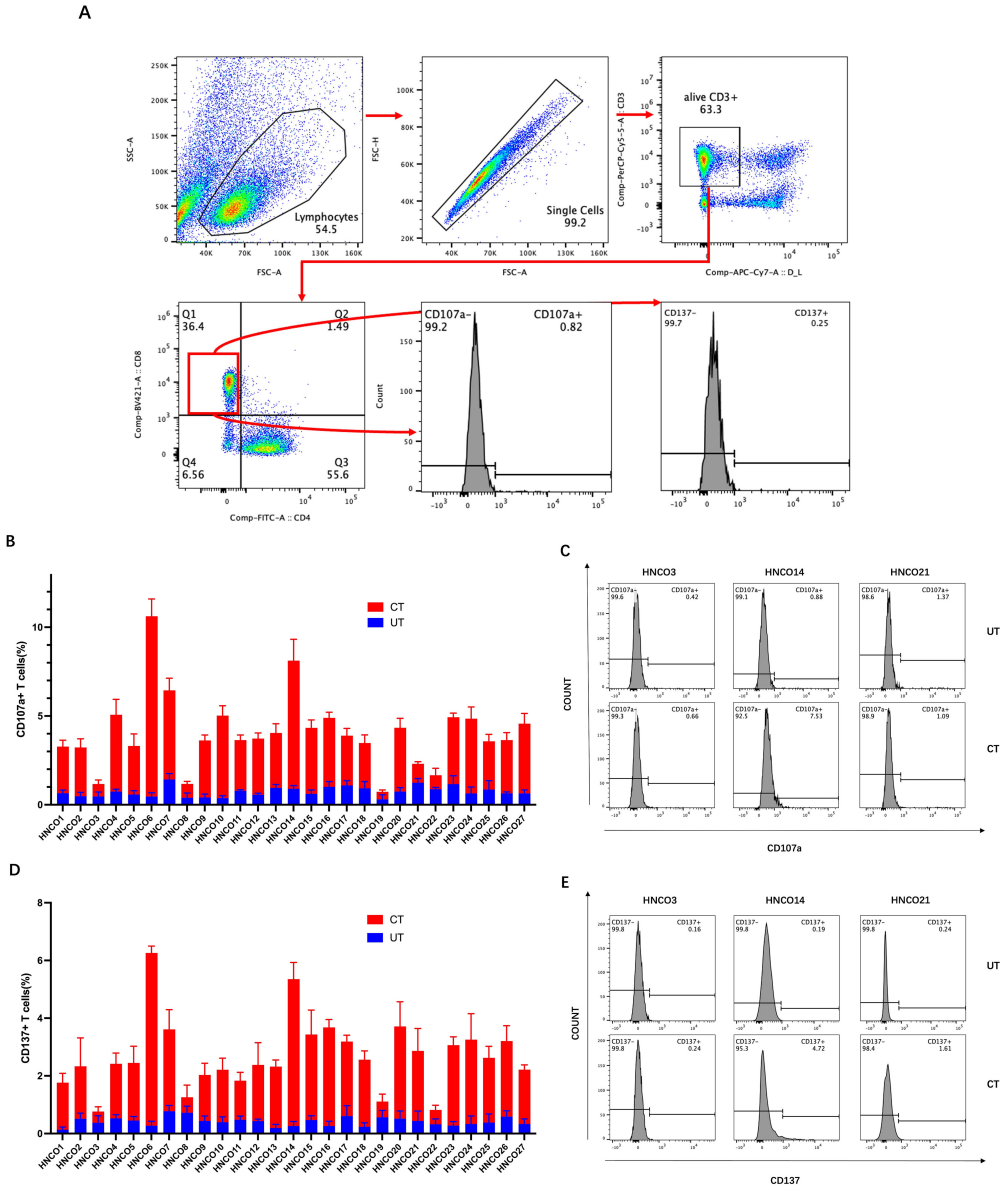
**(A)** Flow cytometry plots of CD107a and CD137 expression on CD8+ T cells. **(B)** Percentage of CD107a-positive cells after co-culture with 27 HCNOs. **(C)** Percentage of CD107a-positive cells after co-culture with HNCO3, HCNO14, and HCNO21. **(D)** Percentage of CD137-positive cells after co-culture with 27 HCNOs. **(E)** Percentage of CD137-positive cells after co-culture with HNCO3, HCNO14, and HCNO21. HNCO, head and neck cancer organoid; CD, cluster of differentiation.

Activated and differentiated CD8^+^ T cells express specific markers, such as CD107a and CD137. Therefore, flow cytometry was used to assess the degranulation and cytotoxicity of CD107a and CD137 expression on CD8^+^ T cells after co-culture with HCNOs (**Figure 5A**).

Consistent with the ELISA results, CD107a and CD137 expression was upregulated in CD8^+^ T cells after co-culture with 22 patient-derived HCNOs, except for HNCO21, where no significant increase in CD107a expression was observed. Furthermore, CD107a and CD137 expression in CD8^+^T cells remained unchanged after co-culture with the remaining four HCNOs (**Figures 5B–E**).

**Figure 6:**
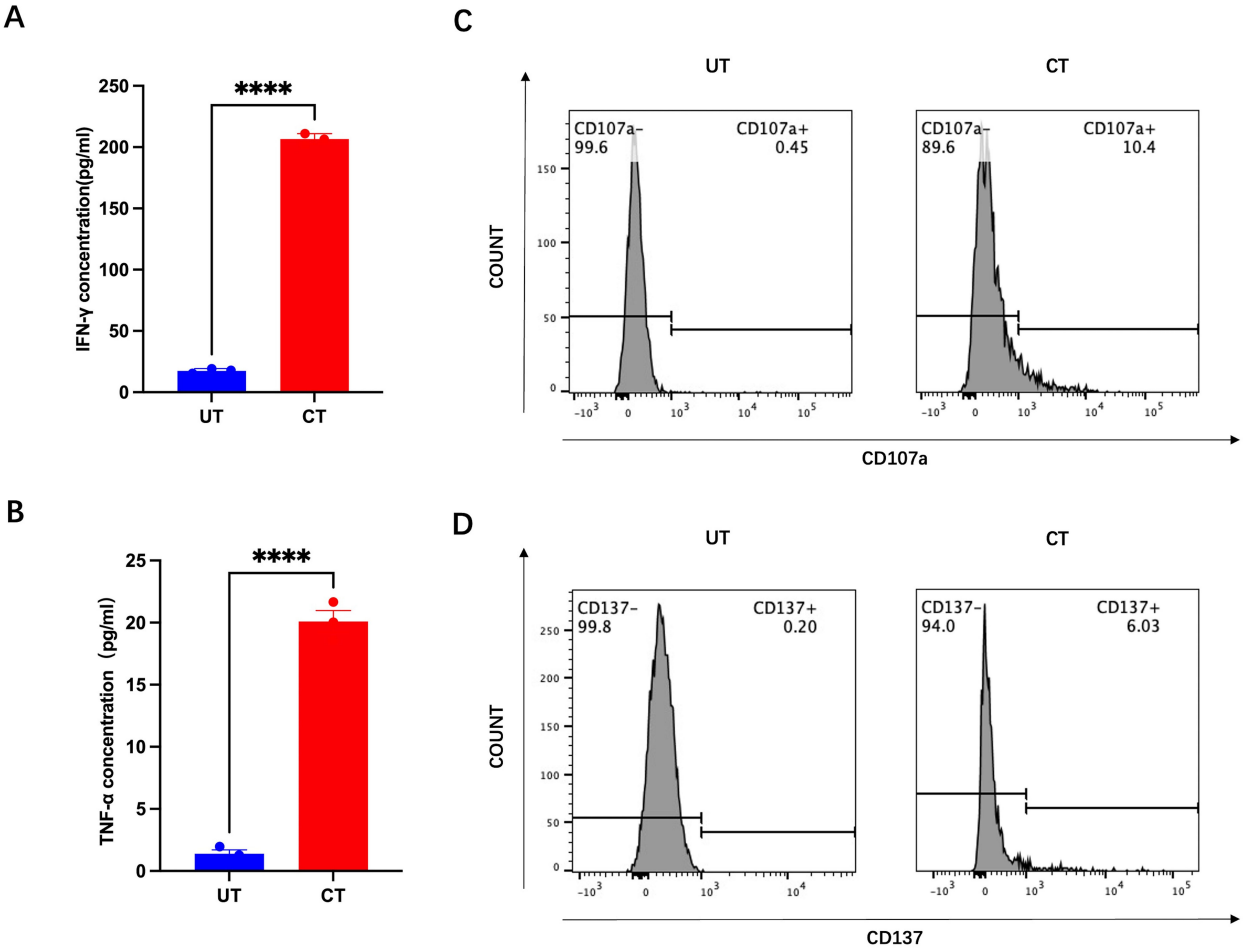
IFN-g **(A)**, TNF-a **(B)**, CD107a **(C)**, and CD137 **(D)** expression before and after co-culture with HNCO6, ****P<0.0001. HNCO, head and neck cancer organoid; CD, cluster of differentiation; IFN-y, interferon-gamma; and TNF-a, tumor necrosis factor alpha.

In some patient samples, CD8^+^ T cells demonstrated enhanced tumor reactivity and cytotoxicity after 2 weeks of co-culture. For instance, in HNCO6, the mean IFN-γ concentration increased from 17.48 pg/mL before to 206.6 pg/mL (approximately 12 times) after 14 days of co-culture (**Figure 6A**). Similarly, the mean TNF-α concentration increased from 1.403 pg/mL before to 20.09 pg/mL after co-culture (approximately 14 times) (**Figure 6B**). Additionally, the percentages of CD107a^+^ cells increased from 0.45% before to 10.4% after co-culture and CD137^+^ CD8^+^ T cells increased from 0.20% before to 6.03% after co-culture (**Figures 6C, D**).

Thus, some HNCOs effectively promote antigen-specific recognition and T-cell activation during co-culture.

### 3.4 Mature HNCOs are killed by induced CD8+ T cells

To determine the cytotoxicity of induced CD8^+^ T cells against autologous mature HNCOs, these cells were co-cultured for 24 h and tumor cell viability was assessed.

**Figure 7:**
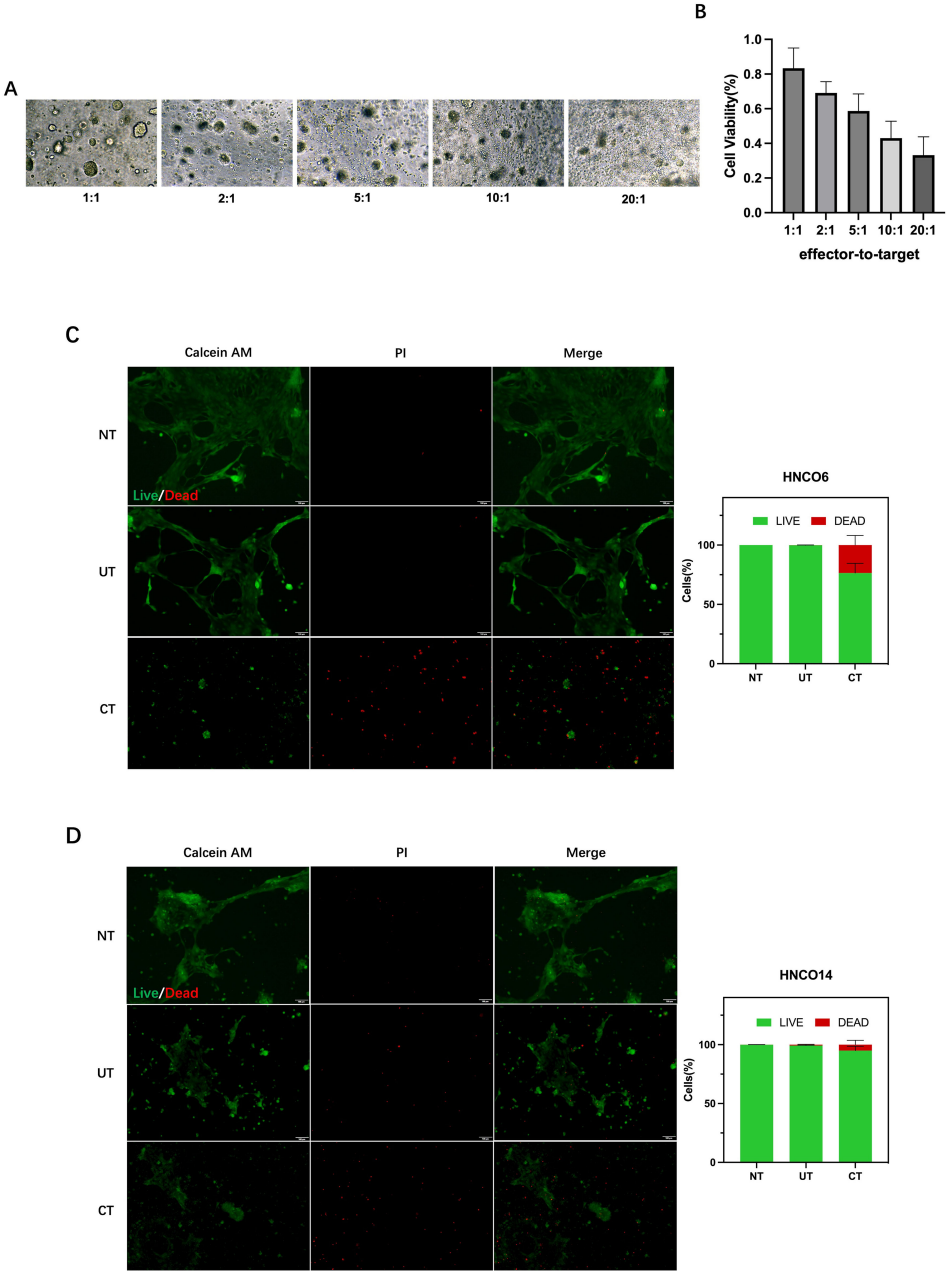
**(A)** HNCO-CD8+ T cell co-culture at various E/T ratios. **(B)** Percentage of HNCO kiling by CD8+ T cels after co-culture at diferent E/ratios measured using the CCK8 assay. **(C)** HNCO6-CD8+ T cell co-culture and celviability. **(D)** HNCO14-CD8+ T cell co-culture andcell viability. HNCO, head and neck cancer organoid; CD, cluster of diferentiation; E/T, effector-to-target; and CCK8, Cell Counting kit-8.

The association between CD8^+^ T cell cytotoxicity and the E/T ratio was assessed by measuring tumor cell viability after 24 h of co-culture at E/T ratios of 1:1, 2:1, 5:1, 10:1, and 20:1. Inverted microscopy indicated pronounced tumor cell lysis with increased E/T ratio (**Figure 7A**). Furthermore, the CCK-8 assay demonstrated that CD8^+^ T cells mediated tumor cell killing at all E/T ratios, with the tumor-killing effect increasing with the ratio (**Figure 7B**).

Tumor killing activity of CD8^+^ T cells was further investigated in the HNCO6 and HNCO14 co-culture models at a 20:1 E/T ratio. Controls comprised media without T cells (NT) and T cells without prior induction (UT). The co-culture of CD8^+^ T cells (CT) with mature HNCOs resulted in 23.5% cell death for HNCO6 (**Figure 7C**) and 5.07% for HNCO14 (**Figure 7D**), significantly surpassing the control groups. Thus, induced CD8^+^ T cells mediate the specific killing of mature HNCOs.

## 4 Discussion

Immunotherapy—particularly immune checkpoint inhibitors—has emerged as a mainstay treatment for cancer. Anti-PD-1 inhibitors are the first-line treatment for HNC, despite low response rates (20%) (4, 27). ACT using tumor-reactive T cells holds promise; however, research is still in the preclinical stages for HNC.

HNC is highly immunosuppressive, characterized by limited TILs. Patients with HNC often lack sufficient tumor-reactive T cells required for ACT (5, 6). Consequently, the *in vitro* culture and expansion of tumor-reactive T cells from PBLs represent a crucial strategy for advancing ACT in HNC because of their accessibility and availability. Moreover, they remain unaffected by the tumor type or volume (28). This study elucidates the feasibility of using HNCOs as a co-culture system for expanding tumor-reactive T cells *in vitro* and evaluating their cytotoxicity.

HNC displays high inter-individual variability, with T cell-mediated tumor killing influenced by various factors, highlighting the potential of personalized cancer treatment (15). Organoids preserve the heterogeneity of the parental tumors and replicate the tumor microenvironment, making them valuable models for personalized therapy. In this study, personalized HNCO-T cell co-culture models were established by pairing organoids with autologous peripheral blood T cells. HNCOs provide a personalized *in vitro* model for studying tumor-T cell interactions and assessing patient sensitivity to immunotherapy (29).

Of the 27 HNCO-T cell co-culture models established, T cells from 22 patients were successfully activated by autologous HNCO single cells, as evidenced by increased cytokine production and upregulated cytotoxicity-associated markers. *In vitro* validation using two HNCO co-culture models confirmed enhanced T cell-mediated cytotoxicity. However, the antitumor response of tumor-reactive T cells obtained using this model may exhibit variability, and the overall success rate remains uncertain. Additionally, the potential for tumor immunosuppression during this cell therapy warrants further investigation.

This study presents a novel approach and experimental foundation for expanding effector cells for ACT in HNC, despite unclear clinical efficacy. The model entirely recapitulates neither TILs or the tumor microenvironment nor their impact on T cell activation and cytotoxicity during ACT, such as immune checkpoint inhibition, which may generate different clinical outcomes. Additionally, the use of non-humanized materials, such as Matrigel—a murine-derived complex of the extracellular membrane with batch-to-batch variation—may affect experimental repeatability and stability, lowering the predictive accuracy of immunotherapy sensitivity assay in clinical settings (30).

Whereas this study elucidates the cytotoxicity of T cells *in vitro*, it remains elusive whether these cytotoxic T cells can be successfully enriched in HNC lesions and directly kill tumor cells after reinfusion. Moreover, the cell preparation process did not adhere to Good Clinical Practice standards for clinical application, warranting further research using animal models.

ACT is a key focus in immunotherapy with substantial implications for cancer treatment. Standardizing the T cell culture protocol is essential for clinical translation, facilitating the re-infusion of successfully induced T cells into patients and providing a novel immunotherapy option for HNC.

## Data Availability

The raw data supporting the conclusions of this article will be made available by the authors, without undue reservation.

## References

[1] Siegel RL Giaquinto AN Jemal A. Cancer statistic. CA: A Cancer J Clin. (2024) 74:12–49. doi: 10.3322/caac.21820 38230766

[2] Sung H Ferlay J Siegel RL Laversanne M Soerjomataram I Jemal A. Global cancer statistics 2020: GLOBOCAN estimates of incidence and mortality worldwide for 36 cancers in 185 countries. CA: Cancer J Clin. (2021) 71:209–49. doi: 10.3322/caac.21660 33538338

[3] Johnson DE Burtness B Leemans CR Lui VWY Bauman JE Grandis JR. Head and neck squamous cell carcinoma. Nat Rev Dis Primers. (2020) 6:92. doi: 10.1038/s41572-020-00224-3 33243986 PMC7944998

[4] Economopoulou P Agelaki S Perisanidis C Giotakis EI Psyrri A. The promise of immunotherapy in head and neck squamous cell carcinoma. Ann Oncol. (2016) 27:1675–85. doi: 10.1093/annonc/mdw226 27380958

[5] Ferris RL. Immunology and immunotherapy of head and neck cancer. J Clin Oncol. (2015) 33:3293–304. doi: 10.1200/JCO.2015.61.1509 PMC4586169 26351330

[6] Moskovitz JM Ferris RL. Tumor immunology and immunotherapy for head and neck squamous cell carcinoma. J Dental Res. (2018) 97:622–6. doi: 10.1177/0022034518759464 PMC6728550 29489423

[7] Wu R Forget M-A Chacon J Bernatchez C Haymaker C Chen JQ. Adoptive T-cell therapy using autologous tumor-infiltrating lymphocytes for metastatic melanoma: current status and future outlook. Cancer J (Sudbury Mass.). (2012) 18:160–75. doi: 10.1097/PPO.0b013e31824d4465 PMC3315690 22453018

[8] Guedan S Ruella M June CH. Emerging cellular therapies for cancer. Annu Rev Immunol. (2019) 37:145–71. doi: 10.1146/annurev-immunol-042718-041407 PMC7399614 30526160

[9] Neelapu SS Locke FL Bartlett NL Lekakis LJ Miklos DB Jacobson CA. Axicabtagene ciloleucel CAR T-cell therapy in refractory large B-cell lymphoma. New Engl J Med. (2017) 377:2531–44. doi: 10.1056/NEJMoa1707447 PMC5882485 29226797

[10] Wang M Munoz J Goy A Locke FL Jacobson CA Hill BT. KTE-X19 CAR T-cell therapy in relapsed or refractory mantle-cell lymphoma. New Engl J Med. (2020) 382:1331–42. doi: 10.1056/NEJMoa1914347 PMC7731441 32242358

[11] Watermann C Pasternack H Idel C Ribbat-Idel J Brägelmann J Kuppler P. Recurrent HNSCC harbor an immunosuppressive tumor immune microenvironment suggesting successful tumor immune evasion. Clin Cancer Res. (2021) 27:632–44. doi: 10.1158/1078-0432.CCR-20-0197 33109740

[12] Canning M Guo G Yu M Myint C Groves MW Byrd JK. Heterogeneity of the head and neck squamous cell carcinoma immune landscape and its impact on immunotherapy. Front Cell Dev Biol. (2019) 7:52. doi: 10.3389/fcell.2019.00052 31024913 PMC6465325

[13] Peltanova B Raudenska M Masarik M. Effect of tumor microenvironment on pathogenesis of the head and neck squamous cell carcinoma: A systematic review. Mol Cancer. (2019) 18:63. doi: 10.1186/s12943-019-0983-5 30927923 PMC6441173

[14] Cattaneo CM Dijkstra KK Fanchi LF Kelderman S Kaing S van Rooij N. Tumor organoid – T cell co-culture systems. Nat Protoc. (2020) 15:15–39. doi: 10.1038/s41596-019-0232-9 31853056 PMC7610702

[15] Choi SY Shim J Gu D Kim SY Kim HJ Shin D-Y. Clonal evolution of long-term expanding head and neck cancer organoid: Impact on treatment response for personalized therapeutic screening. Oral Oncol. (2023) 146:106571. doi: 10.1016/j.oraloncology.2023.106571 37741019

[16] Dijkstra KK Cattaneo CM Weeber F Chalabi M van de Haar J Fanchi LF. Generation of tumor-reactive T cells by co-culture of peripheral blood lymphocytes and tumor organoids. Cell. (2018) 174:1586–1598.e12. doi: 10.1016/j.cell.2018.07.009 30100188 PMC6558289

[17] Driehuis E Kretzschmar K Clevers H. Establishment of patient-derived cancer organoids for drug-screening applications. Nat Protoc. (2020) 15:3380–409. doi: 10.1038/s41596-020-0379-4 32929210

[18] Jacob F Salinas RD Zhang DY Nguyen PTT Schnoll JG Wong SZH. A patient-derived glioblastoma organoid model and biobank recapitulates inter- and intra-tumoral heterogeneity. Cell. (2020) 180:188–204.e22. doi: 10.1016/j.cell.2019.11.036 31883794 PMC7556703

[19] Bhat P Leggatt G Waterhouse N Frazer IH. Interferon-γ derived from cytotoxic lymphocytes directly enhances their motility and cytotoxicity. Cell Death Dis. (2017) 8:e2836. doi: 10.1038/cddis.2017.67 28569770 PMC5520949

[20] Manohar SM. At the crossroads of TNF α Signaling and cancer. Curr Mol Pharmacol. (2023) 17:1–20. doi: 10.2174/1874467217666230908111754 37691196

[21] Aktas E Kucuksezer UC Bilgic S Erten G Deniz G. Relationship between CD107a expression and cytotoxic activity. Cell Immunol. (2009) 254:149–54. doi: 10.1016/j.cellimm.2008.08.007 18835598

[22] Etxeberria I Glez-Vaz J Teijeira Á Melero I. New emerging targets in cancer immunotherapy: CD137/4-1BB costimulatory axis. ESMO Open. (2019) 4:e000733. doi: 10.1136/esmoopen-2020-000733 PMC7333812 32611557

[23] Krzewski K Gil-Krzewska A Nguyen V Peruzzi G Coligan JE. LAMP1/CD107a is required for efficient perforin delivery to lytic granules and NK-cell cytotoxicity. Blood. (2013) 121:4672–83. doi: 10.1182/blood-2012-08-453738 PMC3674668 23632890

[24] Zapata JM Perez-Chacon G Carr-Baena P Martinez-Forero I Azpilikueta A Otano I. CD137 (4-1BB) signalosome: complexity is a matter of TRAFs. Front Immunol. (2018) 9:2618. doi: 10.3389/fimmu.2018.02618 30524423 PMC6262405

[25] Olivo Pimentel V Yaromina A Marcus D Dubois LJ Lambin P. A novel co-culture assay to assess anti-tumor CD8+ T cell cytotoxicity via luminescence and multicolor flow cytometry. J Immunol Methods. (2020) 487:112899. doi: 10.1016/j.jim.2020.112899 33068606

[26] Ferris RL Blumenschein G Fayette J Guigay J Colevas AD Licitra L. Nivolumab for recurrent squamous-cell carcinoma of the head and neck. New Engl J Med. (2016) 375:1856–67. doi: 10.1056/NEJMoa1602252 PMC5564292 27718784

[27] Jenkins RW Barbie DA Flaherty KT. Mechanisms of resistance to immune checkpoint inhibitors. Br J Cancer. (2018) 118:9–16. doi: 10.1038/bjc.2017.434 29319049 PMC5765236

[28] Wei T Leisegang M Xia M Kiyotani K Li N Zeng C. Generation of neoantigen-specific T cells for adoptive cell transfer for treating head and neck squamous cell carcinoma. OncoImmunology. (2021) 10:1929726. doi: 10.1080/2162402X.2021.1929726 34104546 PMC8158031

[29] Zhou Z van der Jeught K Li Y Sharma S Yu T Moulana I. A T cell-engaging tumor organoid platform for pancreatic cancer immunotherapy. Advanced Sci. (2023) 10:2300548. doi: 10.1002/advs.202300548 PMC10427404 37271874

[30] Neal JT Li X Zhu J Giangarra V Grzeskowiak. CL Ju. J. Organoid modeling of the tumor immune microenvironment. Cell. (2018) 175(7):1972–88. doi: 10.1016/j.cell.2018.11.021 PMC6656687 30550791

